# Development and Preliminary Validation of the Scale for Evaluation of Psychiatric Integrative and Continuous Care—Patient’s Version

**DOI:** 10.3389/fpsyt.2017.00162

**Published:** 2017-08-31

**Authors:** Yuriy Ignatyev, Jürgen Timm, Martin Heinze, Sonja Indefrey, Sebastian von Peter

**Affiliations:** ^1^Department of Psychiatry, Psychotherapy and Psychosomatics, Immanuel Klinik, Brandenburg Medical School Theodor Fontane, Rüdersdorf, Germany; ^2^Biometry Section, Competence Center for Clinical Trials, University of Bremen, Bremen, Germany; ^3^Department of Psychiatry and Psychotherapy, Charité University Medicine Berlin, Berlin, Germany

**Keywords:** cross-sectoral mental health care, service users, validation, psychometric measurement, case management, home treatment, interdisciplinary professional practice

## Abstract

This pilot study aimed to evaluate and examine an instrument that integrates relevant aspects of cross-sectoral (in- and outpatients) mental health care, is simply to use and shows satisfactory psychometric properties. The development of the scale comprised literature research, held 14 focus groups and 12 interviews with patients and health care providers, item-pool generation, content validation by a scientific expert panel, and face validation by 90 patients. The preliminary scale was tested on 385 patients across seven German hospitals with cross-sectoral mental health care (CSMHC) as part of their treatment program. Psychometric properties of the scale were evaluated using genuine and transformed data scoring. To check reliability and postdictive validity of the scale, Cronbach’s α coefficient and multivariable linear regression were used. This development process led to the development of an 18-item scale called the “Scale for Evaluation of Psychiatric Integrative and Continuous Care (SEPICC)” with a two-point and five-point response options. The scale consists of two sections. The first section assesses the presence or absence of patients’ experiences with various CSMHC’ relevant components such as home treatment, flexibility of treatments’ switching, case management, continuity of care, cross-sectoral therapeutic groups, and multidisciplinary teams. The second section evaluates the patients’ opinions about these relevant components. Using raw and transformed scoring resulted into comparable results. However, data distribution using transformed scoring showed a smaller deviation from normality. For the overall scale, the Cronbach’s α coefficient was 0.82. Self-reported experiences with relevant components of the CSMHC were positively associated with the patients approval of these components. In conclusion, the new scale provides a good starting point for further validation. It can be used as a tool to evaluate CSMHC. Methodologically, using transformed data scoring appeared to be preferable because of a smaller deviation from normality and a higher reliability measured by Cronbach’s α.

## Introduction

Cross-sectoral mental health care (CSMHC) in Germany provides care to patients with severe mental disorders (1). The advantage of using CSMHC teams lies in their ability to provide the appropriate level of care depending on the patient’s needs. Although CSMHC programs are effective (2), evaluation studies assessing program implementation rarely include a discussion of the implementation measures’ validity (3).

Imported scales that were developed for the assessment of comparable mental health services such as Assertive Community Treatment (4–6), Crisis Resolution Teams (7), Case Management (8), and Community Mental Health Teams (9–12) are inadequate as the German CSMHC differs in many respects from that in named health services (1).

In addition, nearly all existing tools for assessing specific care models rely exclusively on administrative data, evaluating characteristics of treatment from the health care providers’ perspectives. As a result, they usually do not capture the specific effects and experiences of patients and their kin with treatment programs. Further, among those questionnaires that examine user perceptions, some focus on patient opinions (9), though without registering patient experiences (13–15), while others only evaluate satisfaction with mental health care (16). However, self-rated satisfaction is problematic, as patients’ satisfaction was often correlated with the improvement of symptoms or individual characteristics instead of service features (17).

Thus, there is a need for standardized patient questionnaires that allow the concurrent assessment of patients’ experiences and evaluations, and that are well suited to monitoring the characteristics of service provision. To our knowledge, no such questionnaires have been published in international or national literature. The purpose of the current paper was to develop a new, simply to use, and widely applicable, self-reporting questionnaire that covers both the patients’ experiences and opinions about relevant components of CSMHC. The feasibility of the questionnaire and its scale evaluation as well as first psychometric properties should be investigated, on a preliminary basis using a representative sample of psychiatric patients.

## Materials and Methods

Development and feasibility testing of the scale was part of the preparations for a study on “Evaluation of Care Models based on the Regional Psychiatric Budget acc. §64b, V Book of German Social Law.” Since these care models were new to German psychiatry there was no appropriate questionnaire to ask for patient’s evaluation in this setting thus creating the need for developing an own instrument. The study was approved by the Ethics Committee of Medical Chamber Brandenburg [2016, No. S 7 (a)]. All eligible patients were given a comprehensive description of the study and informed that their participation or refusal would not affect their care. After positive patient decision of participation, the written informed consent was obtained.

The development and biometric evaluation of the scale were carried out in construction and pilot testing phases. The construction of the scale included five steps. Firstly, for generating the items, we examined the scientific literature regarding existing scales for assessment mental health services. The most salient themes from these searches were developed into a topic guide for stage 2. Stage 2 consisted of a qualitative study, using the Grounded Theory Methodology (18) in order to extract relevant components of CSMHC: psychiatric patients, their kin and mental health care workers were asked about their experiences with CSMHC and recurrent themes were used to generate items. Thirdly, the first author (Yuriy Ignatyev) created an item pool and a scientific expert panel which consisted of a psychologist and two psychiatrists assessed the content validity of the scale. The expert panel evaluated the wording and item allocation of the tool. In the fourth step, we evaluated face validity. A group of 90 patients from three German hospitals (Imland Klinik Rendsburg, Psychiatrische Klinik Lüneburg, Immanuel Klinik und Poliklinik Rüdersdorf) experienced in with CSMHC were asked to evaluate each item and to indicate if they felt difficulties in replying to the questions. An item was considered to be adapted or excluded if it was problematic for at least five patients. In the fifth step, selected items were checked for reliability, and then items with at least acceptable Cronbach’s α values were combined to generate the preliminary version of the scale. The biometric evaluation aimed to take specific experience of patient into account as it was documented by part one of the questionnaire. In addition, intra-rater reliability was estimated by ratings for contradictory questions in part two of the questionnaire. The evaluation method was developed *a priori*, i.e., not based on empirical data.

The testing phase was carried out using a cross-sectional design in mental health departments of 7 from 16 German hospitals (Klinikum Itzehoe, Südharz Klinikum Nordhausen, Imland Klinik Rendsburg, Rudolf-Virchow-Klinikum Glauchau, Westenküstenklinikum Heide, Immanuel Klinik und Poliklinik Rüdersdorf, Psychiatrische Klinik Lüneburg) that offer CSMHC, from June to December 2016. The only criterion for the inclusion of any hospital into the research program was the given consent of hospitals administration. The sampling was conducted on the basis of equal patient strata from different care sectors (stations, day hospitals, outpatients’ clinics, and on a number of occasions home treatment). The recruitment process *within* each care sector was based on a randomized design. A study group in each hospital consisted of one or two research doctors/psychologists. The inclusion criteria were: age ≥ 18 years, capacity to provide informed consent, ability to read, and understand German. Patients were excluded if they were involuntarily admitted or if their clinical condition limited comprehension (acute mental disorders, severe mental disability, etc.) as judged by their psychiatrist. To assess current psychopathology, a short version of the SCL-90-R (19) was used. The questionnaires were filled out by the participants without assistance. Additionally, some socio-demographic and clinical characteristics (gender, age, education status, employment status, family status, and duration of current mental disorder) were obtained.

A case number of 300 patients was calculated for the preliminary testing of the questionnaire and the biometric method to analyze it. This sample size was calculated to be efficient to detect effect sizes of about 0.333 between two groups of 150 patients or a correlation coefficient of 0.16 between two measures of each patient as significant with α = 5% and power = 80% which seemed convenient for a feasibility study. A *p* > 5% and <10% were thought to represent a trend toward significance.

Common descriptive statistics (count, mean, SD, min, max, and median) were computed for all examined variables. No imputation for missing ratings was conducted as the rate of missing values was less than 5%. The heterogeneity of the responses to specific items was estimated as a quotient of the theoretical variance of random response (equally distributed) by the empirical variance. With respect to the experience questions only affirmative answers (YES) were taken into account as relevant. Both missing affirmative and negative (YES and NO) answers were interpreted as “no experience.”

In order to be able to use the developed ratings as dependent variables for later CSMHC studies, the shape of the resulting distribution of total ratings was checked by estimating skewness and kurtosis and their SDs (20). The relevancy of both statistics was inspected by comparing the quotient of their value by their SD with the numeric value 2. Such procedure has to be interpreted carefully in case of greater deviation from normality (21), however. In our scale, patients are asked to rate therapeutic settings independently of their concrete own experience with these items which is asked for, too. This procedure reflects the fact that patient’s opinions about therapeutic settings have a variety of sources such as social contacts and communication with other patients, friends, family, physicians, and media. Patient’s opinions affect the therapy decisions and efficacy in a positive or negative way. Thus, they should be regarded even if there is a lack of concrete experience. We expected, however, that both reliability and validity of such ratings is lower in comparison with opinions of more experienced patients. To perform both calibration and validation, an assessment of uncertainty in both the data and the instrument is needed. For this purpose, *a priori* rating transformation and weighting was performed. As an accurate calibration of the scale regarding patient competencies could be difficult and there remains some amount of uncertainty we used a sensitivity analysis (22) to evaluate this procedure. Thus, in order to examine psychometric properties of the scale, both data sets (raw and transformed ratings) were analyzed and compared. Results were interpreted as a sensitivity analysis with exploratory character.

For practical reasons, the estimation of Cronbach’s α internal reliability coefficient for only the opinion section of the scale was performed. A Cronbach’s α between 0.6 and 0.7 is considered an acceptable value. A value between 0.7 and 0.9 is a good value, and a value of 0.9 or higher indicates excellent reliability (23). To examine postdictive validity of the experience scale with respect to the opinion rating scale, a multiple linear regression analysis using demographic and clinical characteristics (gender, age, education status, current psychopathology, and duration of current mental disorder) was conducted. According to Cohen’s guidelines, *f*2 ≥ 0.02, *f*2 ≥ 0.15, and *f*2 ≥ 0.35 represent small, medium, and large effect sizes, respectively (24, 25). The statistical computing was performed using SYSTAT 12.0 and nQuery + nTerim 2.0.

## Results

### Developing Phase

#### Items Generation

23 papers were identified in which relevant components of innovative mental health care were explored. We did not find any literature on assessment scales for cross-sectoral mental health care. Nevertheless, a total of 12 papers were found on the relevance for covering assessment aspects for item generation addressing cross-sectoral mental health care. In total, eight papers were included. This search enabled the authors to identify salient concepts in order to produce a topic guide for the qualitative step of development of the scale.

To conduct the qualitative part of the study, two authors (Yuriy Ignatyev and Sebastian von Peter) were as guests in all mental hospitals included in the study. Fourteen focus groups and 12 interviews with mental health care providers were carried out. Additionally, 16 patients were interviewed. Interviews followed a semi-structured format that allowed interviewers to ask spontaneous questions that addressing individual experiences and opinions. On the basis of focus groups and interviews, an item pool was created that consisted of 9 questions relating to the patients’ experiences with CSMHC and 30 questions relating to their evaluation of these experiences. The questions involved a wide range of themes such as home treatment, outpatient treatment, flexibility of treatments’ switching, case management, cross-sectoral treatment groups’ offering, involvement of relatives in the treatment, freely control of therapeutic measures, and interdisciplinary professional practice.

Experts did not suggest any changes of the experience section of the scale. However, they noted that two items from the evaluation section should be removed, as they did not relate to the concept of the scale. Examples of items removed included “*It is good when patients are able to seamlessly transfer between wards of treatment areas*.” It was suggested that four items were in need of rewording as they may be too difficult to understand. Examples included “*I get better quickly when I can share the same space with patients from other treatment areas*,” which was replaced by “*It is good when outpatients, inpatients, and day patients are cared in the same space*.” No additional items were suggested but one expert did comment that a four-point Likert scale for assessing evaluations may be insufficient; the range was changed accordingly to a five-point scale, ranging from 0 “strongly disagree” to 4 “strongly agree.” Expert panel discussions resulted in a scale with the experience section from 9 items and the opinion section included 24 items. To control of careless responses (26), eight of opinion items were worded negatively.

Based on the patients’ viewpoints, six items of the evaluative section were removed due to difficulties of comprehension. Examples included “*Overlapping competencies among staff from different professions lead to competition and are detrimental to me*.” Moreover, patients identified four redundant items and suggested that these items needed rewording. Examples included rewording “*Even patients that are acutely ill can receive home treatment*” to “*Acute patients could also be treated at home* (*i.e*., *home treatment*).” Two items were suggested as in need of clarifying. For example, “*If I have to change my status* (*i.e*., *as an inpatient or day patient or outpatient*), *it is important that I have someone who can guide me through the different treatment areas*.” was amended to “*If I have to change my status* (*i.e*., *as an inpatient or day patient or outpatient*), *it is important that I have someone who can guide me through the different treatment areas and coordinates my treatment*.” The eight items of the experience section were removed due to the poor reliability. The rest of eight items that had at least acceptable reliability were then grouped in the preliminary version of the section (see Supplementary Material). The section included also two negatively worded control opinion items (R5 and R8). In concordance with the reduction of the opinion section, the experience section was also reduced to five items. The domains involved in the whole scale were: current treatment setting (one experience item: E1with four subitems E1a, E1b, E1c, E1d), home treatment (two experience items: E7 and E8, two opinion items: R4 and R9), case management (two experience items: E3 and E4; two opinion items: R2 and R7), cross-sectoral treatment groups’ offering (one experience item: E5, one opinion item: R3), flexibility of treatments’ switching (one experience item: E2, two opinion items: R1 and R6), and interdisciplinary professional practice (one experience item: E6, one opinion item: R10). The scale concerns complex health care system and therefore some overlaps between content domains could be recorded. For example, the opinion item R6 (flexibility of treatments’ switching) was related not only to experience item E2 but also showed overlapping with experience items E3 und E4 (case management).

#### Transformation and Weighting

The construction of the questionnaire resulted into eight items within the experience section, called E1–E8 (see Supplementary Material) in the following. With the exception of E1, each of these items requires a YES or NO answer. E1 is dedicated to the current setting and divided up into four subitems (E1a, E1b, E1c, E1d) to be answered with YES or NO, too. In two cases (E3/E4 and E7/E8) two items cover the same domain of experience, the first addresses this experience in a general and the second in a more meticulous way. For example, E7 asks for experience with home treatment and E8 if this experience was longer than 1 week. A score measuring total experience with situations addressed in this questionnaire was defined as the count of all E-items answered with YES.

The 10 items of the opinion section (R1–R10) have to be answered by a range from “strongly agree” to “strongly disagree” (see Supplementary Material), coded as 0–4. Specific experience is accessible for each of these ratings by experience items: The evaluative items R1, R3, R5, R8, and R10 correspond to items E2, E5, E6, E5, and E6 of the experience section, they cover the same domains. Items R2, R4, R6, R7, and R9 correspond to the doubled items E3/E4, E7/E8, E3/E4, and E7/E8. This correspondence between opinion ratings and experiences was used to develop a weighting of the ratings. For evaluation by a sensitivity analysis, we applied weights to the opinion rating scores with respect to the patient’s corresponding experience. This weighting is documented in Table 1.

**Table 1:** Weighting of opinion values using patient responses to corresponding experience questions.

	Experience question			Weight defined
	Single question^a^	Two sequent questions^b^		
		First question	Second question	
Response to experience question	YES^c^	YES	YES	1
		YES	Not YES^d^	0.75
	Not YES	Not YES	Not YES	0.5
		Missing^e^	YES	0.25
		NO^f^	YES	0

*Corresponding experience (E) and opinion (R) questions*.

*^a^Item: E2 (R1), E5 (R3), E5 (R8), E6 (R5), and E6 (R10)*.

*^b^Item: E3 (R2), E3 (R7), E3 (R6), E4 (R2), E4 (R6), E4 (R7), E7 (R4), E7 (R9), E8 (R4), and E8 (R9)*.

*^c^YES, presence of experience*.

*^d^Not YES, absence of experience or missing response*.

*^e^Missing, missing response*.

*^f^NO, absence of experience*.

The rows present different possible experience answers. Column 1 is dedicated to single experience items, columns 2 and 3 to combined experiences (first item general experience and second item more specific). The fourth column contains the weights as concrete values (default) being used in the sensitivity analysis reported below. The default weights were taken as 1, if the full corresponding experience was documented and positive values below 1 if not. Only the logic contradiction answering YES for the second question and NO for the first question of the same experience were weighted by zero.

The questionnaire allows a quantification of intra-rater reliability of patients’ responses by two pairs of contradictory items (R8 contradicting R3 and R5 contradicting R10). R3 and R10 are directed in favor of the CSMHC program intention, R8 and R5 opposite. If a rating X is documented for one of these pair items, the opposite item should be rated as difference between 4 and X in order to proof full consistency. For example, if R3 = 4 (“strongly agree”), then R8 = 0 (“strongly disagree”) would be a fully consistent reply. Contradicting responses to these items may be a hint for various factors that might reduce the patient’s reliability, such as problematic understanding, distraction, exhaustion, or even cognitive dysfunction. The extent of contradiction may be used to quantify the reliability of the patient’s rating. We propose the following grading based on a pair A, B of contradicting opinion scores:

contradiction score with respect to the pair A, B: C (A, B) = abs (4 − (A + B))/4 as (A, B)− and
reliability score with respect to A,B: Rel (A,B) = 1 − C(A;B)
combined reliability score of each patient: Rel = Mean (Rel (R3, R8), Rel (R5, R10))

A combined rating for contradictory ratings is defined by mix (A, B) = (A + 4 − B)/2, where A is the rating of the question directing to the goal of the model study and B the rating of the contradictory question.

The weight *W* (A) for the rating A of a specific patient is then defined by

W(A)=WE(A)*Rel

Following this definition weighted means may be defined for specific sets of rating items and specific patient samples.

### Pilot Testing Phase

#### Sample Characteristics

*N* = 420 patients were identified as potential participants. *N* = 35 rejected participation; *N* = 385 agreed to participate in the study. The sample consisted of 131 males (34.03%) and 254 females (65.97%). Average age of the participants was 42.1 years (range = 21–88 years, SD = 17.79 years, median = 45.0 years). A majority of patients had high levels of education (50.65% high school graduates) and 29.61% of participants worked for income. A majority of 61.26% was living without partner. The mean score of psychopathological symptomatic using was 1.4, SD = 0.8 (range = 0–3.6). The mean duration of current mental disorder was 10.1 years (range = 0–60 years, SD = 11.2 years).

#### Patient Experiences and Opinions

Analyses of patient experiences items E1a–E1d concerning specific aspects of CSMHC experienced at the time of filling the questionnaire revealed that a majority of participants was currently treated in outpatients’ clinics (34.9%) or day hospitals (35.1%). A fourth of the sample (25.1%) was currently in stationary treatment and only 4.9% participants were currently in home treatment. A majority of patients had experiences with flexibility of treatments’ switching (61.8%) and cross-sectoral treatment groups’ offering (57.9%) and more than half of participants (55.5%) had experiences with case management including its intensive form (27.5%). Fewer respondents 40.3% had experiences with interdisciplinary professional practice. Only very few patients (9.6%) were at least once treated at home including the treatment of at least 1 week (6.7%).

The patient opinion rating is presented in the upper part of Table 2. The counts *N* in the table indicate a small rate of missing values (368 of 385 missing, i.e., 4.4%). The means presented show a range from 1.65 (R8) to 3.12 (R2). It should be kept in mind that R5 and R8 are negatively formulated with respect to R10 and R3 and used for consistency check only. A consistent reply would be the difference between R4 and R5 corresponding to R10 and the difference between R4 and R8 corresponding to R3. Substituting these values (1.85 for R5 and 2.35 for R8) yields a smaller range from 1.78 (R4) to 3.19 (R2). The SDs range from 1.07 to 1.35 the corresponding variances from 1.15 to 1.88. The theoretical variance of an equally distributed random variable indicating high heterogeneity of (random) responses is 2.0. The *F*-values of the items (without R5, R8) range from 1.15 to 1.74. Compared with the critical 5%-value of 1.15 the great majority of item may be well interpreted as acceptable heterogeneity although this comparison is not a formal *F*-test (lack of normality assumption).

**Table 2:** Means (*M*) and SD for opinion values using raw and transformed scoring.

Item of the opinion section		R1	R2	R3	R4	R5	R6	R7	R8	R9	R10
Row scoring	Response^a^ number	374	373	373	372	368	372	372	371	373	371
	*M*	2.61	3.19	2.49	1.76	2.15	2.60	3.15	1.65	2.43	2.86
	SD	1.25	1.09	1.25	1.27	1.19	1.32	1.07	1.35	1.22	1.16

Weight		0.59	0.53	0.58	0.40	NA^b^	0.53	0.53	NA	0.40	0.36

Transformed scoring	*M*	2.67	3.22	2.59	1.88	NA	2.59	3.17	NA	2.56	2.37
	SD	1.78	1.85	1.78	1.73	NA	1.68	1.84	NA	1.28	1.78

Difference between transformed and row *M* values		0.06	0.03	0.10	0.12	NA	−0.01	0.02	NA	0.13	−0.49

*^a^The variation in response number is due to the variation in the number of missing responses*.

*^b^NA, not applicable*.

#### Biometric Transformation of Patient Opinion Ratings

As described in Section “Transformation and Weighting,” the scores have been weighted and transformed in two steps. Firstly, on the basis of Table 1 weights and weighted means of opinion scores were calculated. Secondly, the contradictory scores R3 and R8 as well as R10 and R5 were transformed to new scores R3 and R10, respectively, i.e., R3: = mix (R3, R8) and R10: = mix (R10, R5). The results of both these procedures were defined as transformed scores and presented in the lower part of Table 2. The difference of transformed and raw means is small ranging from −0.01 to +0.13 with the exception of R10 (difference of −0.49). A greater difference may be expected for R3 and R10 taking the definition as a mixed rating into account but the transformed R3 differs only by 0.09 from the raw value. The reason for the R10 exception is a greater inconsistency between rating R10 and R4–R5 (1.01) compared with R3 and R4–R8 (0.15).

**Figure 1:**
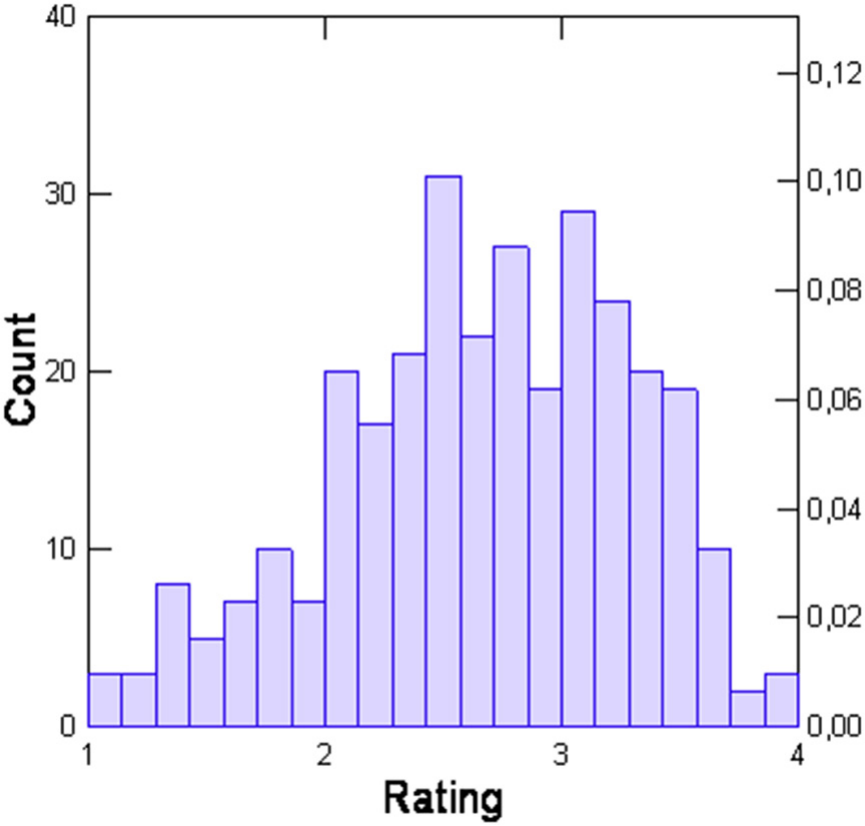
Distribution of transformed total opinion scores of the Scale for Evaluation of Psychiatric Integrative and Continuous Care.

The weighted mean of all opinion ratings calculated for the whole data set on this basis is 2.84 (±0.90). The distribution of these values is demonstrated in Figure 1.

Table 3 showed differences between both row and transformed scoring. There were more cases in the transformed rating due to different handling of failing experience. One would expect a higher SD in the transformed case as there is a mix of experienced and not experienced patient ratings but there is less heterogeneity in the transformed scale (*s* = 0.63 versus *s* = 0.90). Additionally, the skewness and kurtosis are smaller and therefore the feasibility of this version to serve as a depending variable in regression analysis is preferable. The quotient of value and SD of skewness and kurtosis may be compared with 2.00 to detect relevant deviations from normality. These quotients are 2.33 and 6.19 in the case of raw ratings and 2.70, respectively, 1.47 for the transformed ratings.

**Table 3:** Descriptive statistics for total opinion values using raw and transformed scoring.

	*N*^a^	*M*^b^	SD	Skewness	Skewness SD	Kurtosis	Kurtosis SD
Raw scoring	275	2.84	0.90	−0.91	0.15	0.68	0.29
Transformed scoring	307	2.66	0.63	−0.38	0.14	−0.41	0.28

*^a^N, response number. The variation in the response number is due to the variation of missing responses using different scoring*.

*^b^M, mean*.

#### Reliability

As mentioned above, some of the evaluative ratings concern the same content domains: R4 and R9 refer to home treatment, R2 and R7 to case management, and R1 and R6 to flexibility of treatments’ switching. The concordance values for the raw ratings as for case management and treatments’ switching presented in Table 4 were not satisfying as they remain below 0.6. The home treatment rating and the total set of ratings show acceptable α values between 0.6 and 0.7. After biometric transformation all α values were higher compared with the raw values, they are greater as 0.6, i.e., acceptable, and with the exception of treatments’ switching far above the critical value of 0.7 indicating good concordance.

**Table 4:** Cronbach’s α for row and transformed concordant scores of the Scale for Evaluation of Psychiatric Integrative and Continuous Care opinion section (*N* = 385).

Scale domain	Cronbach’s α		
	Item included	Raw score	Transformed score
Home treatment	R4, R9	0.61	0.85
Case management	R2, R7	0.56	0.85
Treatments’ switching	R1, R6	0.55	0.62
Total score	R1–R10	0.66	0.82

The reliability estimated by analysis of contradictory ratings (questions to interdisciplinary professional practice: R3 and R8 and questions to cross-sectoral treatment groups’ offerings: R10 and R5) resulted in a mean value of 0.72 and was incorporated into the weighting process of ratings.

As the total average of ratings may be offered as dependent variable for coming studies in this field this variable was further analyzed in both ways, based on raw ratings and on transformed ratings. For this purpose, raw ratings were only used if the corresponding experience was documented as YES. No adaption of reliability was performed for these raw ratings.

#### Postdictive Validity

A regression analysis (Table 5) was performed for both versions of the total rating. The regression was significant for the transformed rating (*p* = 0.0002, effect size *f*^2^ = 0.160) and had a trend toward significance for the raw rating (*p* = 0.0560, effect size *f*^2^ = 0.075).

**Table 5:** Associations between patients’ characteristics and total opinion value of the Scale for Evaluation of Psychiatric Integrative and Continuous Care using linear regression analyses (*N* = 307).

Patients’ characteristic	Total opinion value using different scoring					
	Raw scoring			Transformed scoring		
	*B*^a^	SE	*p*-Value	*B*	SE	*p*-Value
Experience of the CSMHC	0.16	0.05	<0.01	0.12	0.03	<0.01
Age	0.01	0.01	NS^b^	0.00	0.00	NS
Gender^c^	−0.18	0.15	NS	−0.07	0.09	NS
Education level	−0.09	0.09	NS	−0.09	0.06	NS
Psychopathology level (SCL-K-9 total score)	0.05	0.09	NS	−0.01	0.05	NS
Mental disorder duration (years)	−0.00	0.01	NS	0.00	0.00	NS

*^a^B, unstandardized regression coefficient*.

*^b^NS, not significant*.

*^c^Reference category is female*.

Both regression models proved significant influence of experience. The other factors missed to be significant. This result may be interpreted as a postdictive validity result for the experience evaluation performed here as predictor of the later opinion rating.

## Discussion

### Main Findings

The development process resulted into an 18-item scale with, in the first section a two-point and, in the second section a five-point response options. The first section is related to the patients’ experiences with different relevant components of CSMHC such as home treatment, flexibility of treatments’ switching, case management, cross-sectoral treatment groups’ offerings, and interdisciplinary professional practice. The second section addresses the patients’ opinions regarding these relevant components. Using raw and transformed scoring resulted into comparable results. However, the transformed data showed a smaller deviation from normality and a higher reliability. Therefore, the application of transformed scoring should be preferred.

The developed scale showed a good internal reliability in the measurement of CSMHC. Linear regression analyses demonstrated that the scale has postdictive validity for patient opinions based on their experiences with relevant components of CSMHC. Self-reported experiences with home treatment, flexibility of treatments’ switching, case management, cross-sectoral treatment groups’ offerings, and interdisciplinary professional practice using the Scale for Evaluation of Psychiatric Integrative and Continuous Care (SEPICC) were positively associated with the approval of these mental health care components.

### Strengths and Limitations

To the best of our knowledge, the SEPICC is the first measurement combining differentiated experiences and opinions by patients in regional psychiatric budget hospitals about specific items in relation to the use of CSMHC as part of the treatment program. The tool is brief and simple to use so that it can be applied in mental health care practices. As there is no gold standard for assessing CSMHC, this scale provides a good starting point for further testing and development as well as a pilot scale that can be used in the evaluation of treatment programs. However, the lack of a gold standard metric limits our understanding of the concurrent validity of our tool. Secondly, due to the cross-sectional design of the present study and practical reasons, we were not able to evaluate the test–retest reliability of the scale. Future studies should assess other psychometric properties of the SEPICC such as discriminant validity, construct validity, and criterion validity using administrative records, provider’s perception as well as its measurement invariance across different patient groups. Our research group plans to test for divergent validity of the SEPICC using comparison with a scale measuring general patients’ satisfaction without health service specification. Thirdly, on the basis of our sensitivity analysis the best results were obtained by using transformed scoring that is different from the traditional raw scoring. However, results from both traditional and transformed scoring largely coincided. Fourthly, the survey was conducted at only seven hospitals, raising the issue of the findings’ generalizability. Finally, the majority of the patients in the present study were relatively highly educated. In future studies, it would be necessary to examine the psychometric properties of the SEPICC in patients with different levels of education.

### Comparison against the Literature

The comparison of domains which are specific for our scale and analogous international instruments reflects differences between relevant components of mental health care configurations. Whereas several key principles of Assertive Community Treatment such as *holistic approach to services, integrated services, continuity of care, delivery of services in the community*, and *multidisciplinary team* (27) are well incorporated in the German CSMHC, other features, like *full responsibility for treatment services, high frequency of contact to patients* are less common in Germany (1).

In many instances, our findings replicate previous studies conducted on the basis of self-reported scales, which showed good criterion validity regarding different relevant components of Assertive Outreach Teams (28), Assertive Community Treatment (29), Crisis Resolution Teams (30), Case Management (31), Disease Management (32), and Community Mental Health Teams (33). Remarkably, existing studies identified primarily positive associations between some experiences with innovative mental health care and patient satisfaction. Whereas existing self-reported satisfaction scales focus on individual treatment aspects such as continuity of care (13, 14), interdisciplinary treatment (34), or communication (9), our tool comprises in short form different dimensions of mental health care, which can be presented as total score.

Several authors (17) reported that psychopathology may account for 3–28% of the variance in patient ratings depending on the specific sample and treatment setting. Another study (35) showed that almost 98% of variance in patients’ experiences could be attributed to differences between patients rather than the care unit in which they were treated. For example, younger patients reported significantly less positive perceptions of continuity of care. In another study (36), evidence was found to suggest that satisfaction rates with home treatment were influenced by monthly income and duration of enrollment in the program: individuals with fewer financial resources were in greater need of home care services, hence reporting higher satisfaction, and vice versa. As our scale enabled patients to express their evaluations instead of their degrees of satisfactions, based on the regression analyses, it may be concluded that SEPICC scores may not be dominated by the degree of patient symptom levels or other examined co-variables.

## Conclusion

The SEPICC provides a distinct framework of assessing cross-sectional mental health care, with good reliability, and some satisfactory psychometric properties. Additional studies are needed in order to evaluate the full validity and the true usefulness of the scale in psychiatric research. Because transformed scale scoring showed better statistical assumptions, we would suggest using this scoring in the research practice. The scale can be used in research and routine clinical practice. In research, it could be applied to assess the quality of the CSMHC and provide a basis for advancing knowledge about the critical ingredients of this important service model. In clinical practice, the tool may be used for support and evaluate the service improvement intervention as well as for professional training.

## Ethics Statement

Development and feasibility testing of the scale was part of the preparations for a study on “Evaluation of Care Models based on the Regional Psychiatric Budget acc. §64b, V Book of German Social Law.” The study was approved by the Ethics Committee of Medical Chamber Brandenburg [2016, No. S 7 (a)]. All eligible patients were given a comprehensive description of the study and informed that their participation or refusal would not affect their care. After positive patient decision of participation, the informed consent was obtained.

## Author Contributions

YI, JT, and SP substantial contributions to the conception or design of the work and the acquisition and analysis of the data:. MH and SI interpretation of data for the work. YI and MH drafting the work or revising it critically for important intellectual content.

## Conflict of Interest Statement

The authors declare that the research was conducted in the absence of any commercial or financial relationships that could be construed as a potential conflict of interest.
