# Supplementary material for: Development and Preliminary Validation of the Scale for Evaluation of Psychiatric Integrative and Continuous Care—Patient’s Version
Source: Front Psychiatry. 2017 Aug 31;8:162. doi: 10.3389/fpsyt.2017.00162 (PMC5583144; doi:10.3389/fpsyt.2017.00162)
Supplement: Supplementary file 1 [file data_sheet_1.docx]

Supplementary Material

**Development and Validation of the Scale for Evaluation of Psychiatric Integrative and Continuous Care (SEPICC) – patient’s version**

**Yuriy Ignatyev*, Jürgen Timm, Martin Heinze, Sonja Indefrey, Sebastian von Peter**

*** Correspondence:** Corresponding Author: Y.ignatyev@immanuel.de

**Scale for Evaluation of Psychiatric Integrative and Continuous Care (SEPICC) – patient’s version**

**Section 1.**

| E1. | **Currently** I am being treated as a/an: |  |
| --- | --- | --- |
| a) | outpatient | **YES**  **NO** |
| b) | day patient | **NO**  **YES** |
| c) | inpatient | **NO**  **YES** |
| d) | home treatment patient | **NO**  **YES** |
| E2. | Have you ever experienced different types of patients (i.e. outpatient/day patient/inpatient) being treated in the **same** place? (e.g. an outpatient coming to a doctor on the ward) | **YES**  **NO** |
| E3. | Have you ever received treatment in **different** wards or in a **different** department in this hospital (e.g. you were previously an inpatient and are now an outpatient or day patient)? | **YES**  **NO** |
| E4. | *(Only if you answered ‘Yes’ to Question 3):*  After you changed wards/departments, did you go on to be treated by the same team? | **YES**  **NO** |
| E5. | Have you ever experienced different types of patients (i.e. outpatients/day patients/inpatients) being treated in the **same** group (e.g. outpatients together in the same group as day patients)? | **YES**  **NO** |
| E6. | During your treatment, have you ever experienced that one group of professionals took over the work of another group of professionals? (e.g. a doctor giving you a recommendation in relation to social issues or a nurse/carer carrying out a psychoeducational group?) | **YES**  **NO** |
| E7. | Have you ever been visited by a therapeutic team (i.e. two or more therapists) from the hospital at home (i.e. home treatment)? | **NO**  **YES** |
| E8. | *(Only if you answered ‘Yes’ to Question 7):*  Did the treatment you received at home last longer than one week? | **YES**  **NO** |

**Section 2.**

| No. | Item | strongly disagree | disagree | undecided | agree | strongly agree |
| --- | --- | --- | --- | --- | --- | --- |
| R1. | It is good when outpatients, inpatients and day patients are cared in the **same** space. |  |  |  |  |  |
| R2. | If it came to changing ward or treatment areas (inpatient/day patient/outpatient), it would be important to me that I be treated by the **same** team**.** |  |  |  |  |  |
| R3. | I gain from being treated in therapeutic groups **together** with different types of patients (inpatients/day patients/outpatients). |  |  |  |  |  |
| R4. | Acute patients could also be treated at home (i.e. home treatment). |  |  |  |  |  |
| R5. | It is to my benefit if the professional groups involved in my care limit themselves to their competencies in order to avoid an overlap. |  |  |  |  |  |
| R6. | It is good if patients, after being discharged from a ward, go on to be treated ambulatory on the **same** ward as outpatients, and not in a separate outpatients’ clinic. |  |  |  |  |  |
| R7. | If I have to change my status (i.e. as an inpatient or day patient or outpatient), it is important that I have someone who can guide me through the different treatment areas und coordinates my treatment. |  |  |  |  |  |
| R8. | Therapeutic groups should consist of only one type of patient (i.e. **either** inpatients **or** day patients **or** outpatients) so that they are helpful. |  |  |  |  |  |
| R9. | It is helpful to be treated at home because home treatment incorporates the everyday life of patients. |  |  |  |  |  |
| R10. | It adds value to the therapeutic process if the services offered by doctors, carers/nurses, social workers, psychologists or other professionals overlap and thereby can complete or compensate for each other. |  |  |  |  |  |
